# The Influence of Social Media on Dental Education: Dental Students’ Perspectives

**DOI:** 10.1155/ijod/5103984

**Published:** 2026-10-04

**Authors:** Houria Shokrollahzadeh Behbahani, Rezvane Ghazanfari, Pouyan Aminishakib, Mahya Hasanzade

**Affiliations:** ^1^ Dental Research Center Dentistry Research Institute School of Dentistry Tehran University of Medical Sciences Tehran Iran tums.ac.ir; ^2^ Department of Prosthodontics Dental Research Center Dentistry Research Institute School of Dentistry Tehran University of Medical Sciences Tehran Iran tums.ac.ir; ^3^ Oral and Maxillofacial Pathology Department School of Dentistry Tehran University of Medical Sciences Tehran Iran tums.ac.ir

**Keywords:** dental students, education, social media

## Abstract

**Introduction:**

In recent years, the field of communication has undergone significant transformations, substantially influencing the education of dental students. This study aims to identify and evaluate these influences in order to contribute to the improvement of current learning and educational methods.

**Materials and Methods:**

A cross‐sectional study was conducted. A semistructured questionnaire was developed, and its validity and reliability were assessed. A total of 112 dental students from the University of Tehran were randomly selected, and the final questionnaire was administered manually. Data analysis was performed using SPSS software (version 25).

**Results:**

Students reported longer durations of use for WhatsApp and Telegram compared to Instagram and YouTube, with Instagram ranking second and YouTube third. These differences were statistically significant (*p*  < 0.001). Similarly, daily usage was highest for WhatsApp and Telegram, followed by Instagram and YouTube, with a significant difference observed among platforms (*p*  < 0.001). The influence of educational resources on academic learning was comparable across YouTube, WhatsApp, and Telegram, with no significant difference detected (*p* = 0.128). However, all three platforms demonstrated a significantly greater influence than Instagram, which ranked lowest (*p*  < 0.001).

**Conclusion:**

This study indicates that dental students widely use social media for learning despite its limited integration into formal educational programs, reflecting a generally positive but cautious perspective. While platforms such as YouTube, WhatsApp, and Telegram demonstrate a greater influence on academic learning compared to Instagram, a notable proportion of students do not verify the credibility of scientific content encountered online. Integrating guidance on the effective and critical use of social media into educational strategies, particularly on commonly used platforms such as YouTube, may enhance learning quality. Additionally, training students to critically assess online information is essential to optimize virtual learning outcomes.

## 1. Introduction

The term “social media” refers to systems that enable users to connect and share information with each other. Social media has become a significant component of digital communication, influencing various aspects of society, including communication, marketing, and social interactions. Popular social media platforms include Facebook, Instagram, Twitter, LinkedIn, and others [1]. According to statistical data from 2008 to 2020, 82% of Americans use social media and have personal profiles [2]. The average global user spends ~145 min online per day, up from 142 min in the previous year [3]. Given the large user base and extended usage duration, the social media is expected to have a significant influence.

One notable area influenced by the social media is education. In dentistry, students encounter complex topics that require concentration and deep learning. With the rising adoption of online platforms for educational purposes after the COVID‐19 pandemic, it is important to identify the key factors influencing students’ knowledge and motivation. The aim of this study is to investigate the perspectives of dental students regarding the influence of different social media platforms (WhatsApp, Telegram, YouTube, and Instagram) on their education.

Previous studies have examined social media platforms with different characteristics using identical questionnaires, resulting in limited specificity in findings [4–6]. To address this gap, the present study focuses on different social media platforms by grouping those with similar functions and educational purposes, while analyzing platforms with distinct characteristics separately. To our knowledge, no similar study has been conducted in Iran. This is important as previous studies have shown that social media use among dental students varies across countries.

## 2. Materials and Methods

This study is a cross‐sectional investigation conducted based on a semistructured questionnaire design. The questionnaire was developed based on previously published instruments and relevant literature [4–6], with modifications to address platform‐specific characteristics and to overcome limitations of existing tools in evaluating the social media use among dental students. In the initial phase of the study, ethical approval was obtained from the ethics committee with the code IR.TUMS.DENTISTRY.REC.1401.050 at the Medical Research Center of Tehran University of Medical Sciences.

In the initial design phase, the questionnaire was evaluated by three faculty members from the University of Tehran with expertise in dentistry (two prosthodontists and one oral and maxillofacial pathologist). Two of the reviewers had experience using social media, providing additional practical insight into the study context. After incorporating their feedback, the questionnaire underwent validity and reliability assessment. Content validity was assessed using both qualitative and quantitative methods. The questionnaire items were evaluated by 10 faculty members in terms of relevance, clarity, and appropriateness. Items that did not achieve consensus were revised until agreement was reached. In the qualitative assessment, grammar, wording, and scoring were reviewed by experts. Quantitative evaluation of content validity was performed using the content validity ratio (CVR) and content validity index (CVI). The CVR score was 0.80 and the CVI score was 0.90, indicating acceptable content validity. Face validity was assessed using both qualitative and quantitative approaches. Seven clinical‐year dental students (fourth to sixth year) from the Tehran University of Medical Sciences were asked to complete the questionnaire. In the qualitative phase, items were evaluated for difficulty, ambiguity, and relevance and were revised accordingly. Subsequently, the item influence method was used to quantitatively assess and refine the items. Reliability was evaluated using a test–retest method. The same seven students completed the questionnaire again after a 1‐week interval. Based on the analysis of 14 completed questionnaires, the instrument demonstrated 100% repeatability and reliability.

The finalized questionnaire was distributed manually to the target population, consisting of eligible fourth‐, fifth‐, and sixth‐year dental students at the Tehran University of Medical Sciences. In previous studies, the study population ranged from 200 to 1000 participants [4–6]. In the present study, a census sampling approach was used, and an attempt was made to include the maximum number of eligible students available during the study period. Based on the expected number of eligible students, a minimum sample size of 100 participants was targeted. In total, 112 clinical‐year dental students completed the questionnaire and were included in the analysis.

Following data collection, responses were entered into Microsoft Excel and analyzed using SPSS software version 25. All participants completed the mandatory questionnaire items. Descriptive statistics were reported as frequencies and percentages. For comparisons of ordinal responses across Instagram, WhatsApp/Telegram, and YouTube, the Friedman test was used because the same participants answered comparable questions on each platform. When the Friedman test showed a significant difference, pairwise comparisons were performed using Wilcoxon signed‐rank tests with Bonferroni correction. A *p*  < 0.05 was considered statistically significant.

The final questionnaire consisted of 55 items (54 mandatory and one optional), organized into five sections. The first section collected general information, including consent, a five‐digit anonymization code, age, academic year, and gender. Participants generated a unique five‐digit code to enable anonymous matching of responses (for example, in the test–retest phase). The code was used solely for data management purposes and was not included as a variable in the statistical analysis. The second section assessed the Instagram usage in terms of frequency, purpose, and patterns. The third section evaluated the use of WhatsApp and Telegram, which were analyzed together due to their similar characteristics as messaging‐based platforms used for direct communication, file sharing, and closed educational groups, in contrast to Instagram, which is primarily a visual feed‐based platform, and YouTube, which is video‐based. The fourth section focused on the frequency, patterns, and purposes of YouTube use. The fifth section included concluding questions (Appendix A: Table A1).

## 3. Results

### 3.1. Demographic Characteristics

Among the study participants, the majority (55.4%) were aged 20–25 years, representing the largest age group. This was followed by participants aged 25–30 years (42%) and 30–35 years (2.7%). Out of the 112 dental students included in the study, 69 (61.6%) were female and 43 (38.4%) were male. In terms of academic level, most participants were in their fifth year (46.4%), followed by sixth‐year students (37.5%) and fourth‐year students (16.1%).

### 3.2. Findings on Instagram as a Preferred Social Media Platform

As shown in Figure 1, the majority of dental students identified inaccuracies in learning materials and the risk of addiction as the main drawbacks of using Instagram as an educational tool.

**Figure 1:**
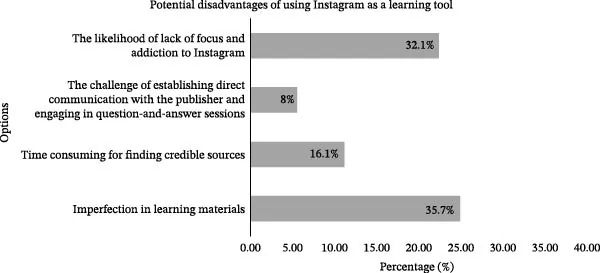
Potential drawbacks of using Instagram in learning.

According to Figure 2, most dental students preferred video‐based posts over other types of educational content available on Instagram.

**Figure 2:**
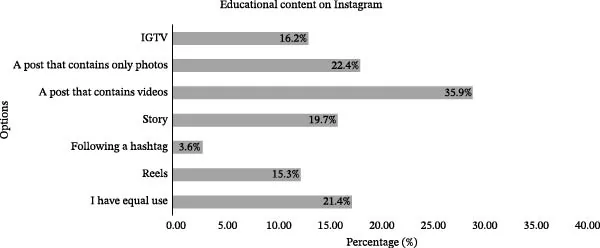
Educational content available on Instagram.

### 3.3. Findings on WhatsApp and Telegram as a Preferred Social Media Platform

According to Table 1, 50.9% of dental students reported using educational channels on Telegram as their primary source of learning materials. Additionally, 80.4% indicated that lecture notes are the most commonly used resources on WhatsApp and Telegram.

**Table 1:** Findings of part three of the questionnaire.

Findings related to WhatsApp & Telegram usage	Percentage (%)
Students using both WhatsApp & Telegram	90.2
Dentistry students using WhatsApp and Telegram for over 5 years	83.9
Average daily usage (hours)	93.8 (2 h)
Average educational usage (h)	82.2 (2 h)
Primary source of educational content (using Telegram educational channels)	50.9
Top reasons for usage: • Communication with friends & acquaintances • Sharing academic resources • Learning educational materials	94.6 80.4 67
Least common reason for usage (job hunting and advertisements)	35.7
Primary use of resources: Most likely: Lecture notes Least likely: Educational images	80.4 2.7
Not searching for official sources of educational content found on WhatsApp and Telegram	58
Evidence‐based nature of educational resources presented on WhatsApp and Telegram	27.7

### 3.4. Findings on YouTube as a Preferred Social Media Platform

As illustrated in Figure 3, 70.6% of dental students reported using YouTube videos to prepare for fixed prosthodontics practical courses, while they were least likely to use such videos for pediatric dentistry preparation.

**Figure 3:**
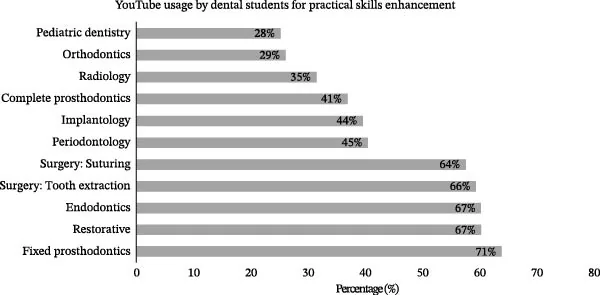
Use of YouTube videos for practical course preparation.

### 3.5. Findings From the Concluding Questions

A majority of students (58.1%) reported that there is no difference between female and male instructors in their use of social media for educational purposes. Additionally, 64.3% of students perceived that instructors who frequently use social media in education are typically under the age of 40. Regarding teaching preferences, 58.% of students favored a hybrid approach that combines online and in‐person learning, while 24.1% expressed a preference for exclusively online classes.

### 3.6. Duration of Social Media Use (in Years)

The results indicate that students reported using WhatsApp and Telegram for a longer duration, in terms of years, compared to other social media platforms. Instagram ranked second, while YouTube was third in terms of duration of use. A statistically significant difference was observed among these platforms with respect to years of usage (*p*  < 0.001).

### 3.7. Daily Use of Social Media

The findings of this study indicate that students most frequently use WhatsApp and Telegram on a daily basis, ranking these platforms first in terms of daily usage. Instagram ranked second, followed by YouTube in third place. A statistically significant difference was observed in the daily use of Instagram, YouTube, WhatsApp, and Telegram among students (*p*  < 0.001).

### 3.8. Influence of Available Resources on Subject Learning via Social Media

The findings indicate that the influence of educational resources available on YouTube, WhatsApp, and Telegram on subject learning was comparable, with no statistically significant difference observed among these platforms (*p* = 0.128). In contrast, these platforms demonstrated a significantly greater influence on subject learning compared to Instagram, which ranked lowest. This difference was statistically significant (*p*  < 0.001).

### 3.9. Evidence‐Based Nature of Educational Resources Presented on Social Media

According to Tables 1–3, 53.6% of students considered instructional videos for practical dentistry courses on YouTube to be evidence‐based. In comparison, 35.7% of students perceived educational content on Instagram as evidence‐based, while only 27.7% held this view for resources shared via WhatsApp and Telegram. Overall, these findings indicate that YouTube is regarded by students as the most reliable and credible source of educational content among the platforms evaluated.

**Table 2:** Findings of part two of the questionnaire.

Findings related to Instagram usage	Percentage (%)
Students using Instagram	92
Dentistry students using Instagram for over 5 years	65.5
Average daily usage (h)	80.4 (2 h)
Students following a professor‐introduced educational page on Instagram	83.9
Educational post reading based on caption length	53.6 (below four sentences)
Students referring to credible sources for new scientific topics found on Instagram	55.4
Top reasons for Instagram usage: • Connecting with friends and acquaintances • Entertainment • Learning educational content	84.8 82.1 74.1
Not searching for official sources of educational content found on Instagram	62.5
Least common reason for Instagram usage (job hunting)	28.6
Evidence‐based nature of educational resources presented on Instagram	35.7

**Table 3:** Findings of part four of the questionnaire.

Findings related to YouTube usage	Percentage (%)
Students using YouTube	87.5
Dentistry students using YouTube for over 5 years	48.2
Weekly YouTube usage	35.7
Daily YouTube usage (<2 h)	18.8
Useful for learning practical courses in dentistry	84.8
Used for preparation of unfamiliar practical work	80.4
Recommendation to classmates for dentistry learning	79.5
Desire for faculty to post educational videos on YouTube	67
Reasons for usage: • Watching educational videos • For fun and entertainment • Watching noneducational videos	45.5 28.6 13.4
Evidence‐based nature of educational resources Presented on YouTube	53.6
Not searching for official sources of educational content found on YouTube	55.4

### 3.10. Search for Official Sources Not Presented on Social Media

According to Tables 1–3, 62.5% of students reported that they did not search for the official source when encountering a scientific article without a citation on Instagram. Similarly, 58% indicated that they do not seek the original source when such content is encountered via WhatsApp and Telegram. In the case of YouTube, 55.4% of students reported not searching for the official source when presented with a scientific article lacking proper attribution. Overall, these findings suggest that more than half of the students do not actively verify or seek official sources when exposed to uncited scientific content across the evaluated platforms.

### 3.11. Percentage of Use for Each Social Media Platform

The findings show that Instagram was the most widely used platform among students (92.0%), followed by WhatsApp and Telegram (90.2%) and YouTube (87.5%). Lower usage rates were observed for Twitter (31.4%), LinkedIn (22.4%), Facebook (20.6%), and TikTok (5.4%).

### 3.12. Students’ Reasons for Using Social Media

According to Tables 1–3, the primary reason for using Instagram among 84.8% of students was to connect with friends and acquaintances. Similarly, 94.6% of students reported using WhatsApp and Telegram mainly for communication with friends and acquaintances. In contrast, 45.5% of students indicated that their main reason for using YouTube was to watch educational videos related to their courses.

Overall, these findings suggest that students primarily use YouTube for academic purposes, whereas Instagram, WhatsApp, and Telegram are predominantly used for social interaction and have comparatively less educational significance.

## 4. Discussions

The majority of participants in this study were aged between 20 and 25 years. Fifth‐year students constituted the largest proportion of the sample, although participants were selected randomly across academic years. This distribution may explain why students reported watching more videos related to fixed prosthodontics as it is a practical course commonly undertaken during the fifth year. In contrast, pediatric dentistry is typically studied in the sixth year, which may account for the lower engagement with related video content.

In 2020, Mona Rajah and colleagues studied the influence of social media on dental students’ learning at three universities in Saudi Arabia, finding WhatsApp, Snapchat, Twitter, and Instagram were most used [4]. A 2019 study at New York University also highlighted YouTube and Google as the top platforms for virtual education among dental students [5]. Previous research lacked a centralized questionnaire due to the varied nature of the social media examined. In 2017, Martina Drahošová and colleagues explored media use in the European Union, identifying Facebook, Google+, Instagram, LinkedIn, and Twitter as the most popular [7]. This study showed differences between Iran and the EU, with LinkedIn being used by 43.2% of the EU population, compared to just 22.4% among dental students in the current study, reflecting variations in platform popularity across countries.

One of the main findings of this study was the distinction between general use and educational preference among the social media platforms. Although Instagram was the most widely used platform among participants and most students indicated that they would follow a professor‐recommended Instagram page for educational purposes, only a small proportion preferred it for academic learning. This may reflect the platform’s entertainment‐oriented nature. Instagram’s short, visually engaging, and fast‐consumed content format may increase user engagement, but it may also limit the depth of learning. Students’ feedback supports this interpretation, as they identified incomplete learning content as a significant educational drawback of the platform. Therefore, Instagram may be better understood as a tool for brief exposure, motivation, and initial familiarization with concepts rather than a primary source for deep learning.

Another potential drawback of using Instagram is its influence on focus and concentration. Students expressed concerns that excessive use could hinder their ability to concentrate on academic materials, potentially leading to distractions and reduced productivity. Studies by Martina Drahošová [7] and Mona Rajeh [4] confirmed that students view time wastage and addiction as the greatest harms of social media [4, 7]. These concerns seem universal across countries, suggesting that they are common issues with using social media as educational tools. To mitigate these effects, students could implement time restrictions.

In contrast, YouTube was identified as the most preferred platform for learning dental subjects, particularly practical courses. This finding is educationally meaningful because dentistry is a highly visual and skill‐based discipline, and video‐based learning naturally aligns with the demonstration of clinical and preclinical procedures. Students reported that they use YouTube to prepare for procedures they have not yet performed and to learn practical courses such as fixed prosthodontics, restorative dentistry, and endodontics. This suggests that YouTube may fill an important gap between theoretical instruction and psychomotor skill acquisition by allowing repeated viewing, flexible pacing, and exposure to different teaching styles. The strong student support for faculty‐produced educational videos on YouTube further highlights the potential of structured video resources in dental education. The majority of students (67%) in this study agree that their dental school should post educational videos for learning practical aspects of dental courses on YouTube. This finding is supported by a study conducted in 2020 by Lorel Burns and colleagues [6], where 89% of students expressed interest in educational video posts by their university on social medias like YouTube.

While YouTube was favored for direct instructional content, WhatsApp and Telegram were widely used for educational exchange, though their perceived role was more organizational than instructional. These platforms were primarily used for peer‐to‐peer communication, sharing lecture notes, and distributing course materials. This suggests that WhatsApp and Telegram function more as collaborative tools than as primary teaching platforms. In other words, while YouTube supports demonstration and visualization of practical skills, Telegram and WhatsApp support peer‐to‐peer learning, coordination, and access to condensed materials. This complementary pattern is important because it indicates that students do not view all social media platforms in the same way; rather, each platform serves a distinct role in the learning ecosystem. These findings align with studies by Sirajul Munir [8] and Hibah Khalid ALadsani [9], which also highlighted the educational potential of these platforms for communication and material sharing rather than formal instruction.

An important concern raised by the present study is the issue of source credibility and evidence‐based learning. Although many students considered YouTube content to be evidence‐based, and some believed the same about Instagram and WhatsApp and Telegram, a considerable proportion of students reported that they do not search for the official source when encountering scientific content without references. This finding has important implications. It suggests that, despite the widespread use of social media platforms for learning, critical appraisal and source verification behaviors may remain insufficient. In a field such as dentistry, where inaccurate or incomplete knowledge can directly affect clinical performance and patient care, this issue is especially significant. Therefore, digital literacy and evidence‐appraisal skills should be reinforced as part of dental education. Students should be trained not only in how to access educational content quickly but also in how to evaluate its scientific quality, identify bias, and confirm its source.

Another notable result was that students preferred a blended learning model, combining online education with face‐to‐face teaching. This finding suggests that students do not perceive social media and online resources as replacements for conventional education but rather as complementary tools that can enrich the learning process. For dental education in particular, where practical training, direct supervision, and feedback are essential, this preference for a blended model seems reasonable. Social media may improve access to materials, repetition of content, and flexibility of learning, while in‐person education remains necessary for supervised practice, correction of mistakes, and professional development.

Dental schools may benefit from adopting a more structured and faculty‐guided approach to social media use in education. Because students are already using these platforms extensively, the question is no longer whether social media should play a role in dental learning but how this role can be optimized. Faculty members could introduce reliable YouTube channels, create short evidence‐based instructional videos, recommend or create trustworthy Telegram channels or WhatsApp groups for academic communication, and teach students how to verify scientific claims encountered online. For Instagram, educational content may be most effective when presented in concise video‐based formats with short captions and clear links to more formal resources. Such an approach may preserve the engagement advantages of social media while reducing the risks of misinformation and superficial learning.

Future research could expand on this study by including participants from multiple institutions and across all academic years rather than being limited to dental students from a single university and only clinical‐year students. This would provide a more comprehensive understanding of social media use and its influence on learning throughout dental education.

Additionally, the role of emerging technologies such as ChatGPT and other AI‐based tools in dental education remains an important area for exploration. As AI tools become more integrated into educational settings, future studies should examine how platforms like ChatGPT influence dental students’ learning experiences, content comprehension, and engagement with educational materials.

## 5. Conclusions

The present study showed that social media is widely used by dental students as a supplementary learning tool, although it is not formally integrated into the dental curriculum. YouTube was the preferred platform for learning academic and practical dental content, while WhatsApp and Telegram were mainly used for communication, sharing lecture notes, and exchanging educational resources. Instagram, despite being the most commonly used platform, appeared less suitable for formal learning.

The findings suggest that social media can support dental education when used in a structured and evidence‐based manner. Dental schools may benefit from guiding students toward reliable online resources, producing faculty‐approved educational videos, and strengthening students’ digital literacy and source‐evaluation skills. These strategies may help improve the educational value of social media while reducing the risk of misinformation in dental learning.

## Author Contributions

**Houria Shokrollahzadeh Behbahani**: conceptualization, formal analysis, investigation, project administration, methodology, writing – original draft, writing – review and editing. **Mahya Hasanzade**: conceptualization, methodology, supervision, writing – review and editing. **Rezvane Ghazanfari**: methodology, writing – review and editing. **Pouyan Aminishakib**: resources, writing – review and editing.

## Funding

No funding was received for this study.

## Disclosure

All authors have read and approved the final version of the manuscript. Mahya Hasanzade, as the corresponding author, had full access to all of the data in this study and takes complete responsibility for the integrity of the data and the accuracy of the data analysis.

## Ethics Statement

Ethical approval was obtained from the ethics committee with the code IR.TUMS.DENTISTRY.REC.1401.050 at the Medical Research Center of Tehran University of Medical Sciences.

## Conflicts of Interest

The authors declare no conflicts of interest.

## Data Availability

The data that support the findings of this study are available from the corresponding author upon reasonable request.

## Appendix A

**Table A1:** Investigating the views of dental students regarding the influence of social media on their education.

Number	Question	Answer
**General questions**		

1	Do you consent to answering this questionnaire for scientific research purposes?	Yes ☐ No ☐
2	Please write a five‐digit code for yourself	‐‐‐‐‐
3	Age	Under 20 years ☐ 20 to 25 years ☐ 25 to 30 years ☐ 30 to 35 years ☐ Over 35 years ☐
4	Gender	Female ☐ Male ☐
5	What academic year are you in?	Fourth ☐ Fifth ☐ Sixth ☐
6	List your most used social medias in order.	1. ……… 2. ……… 3. ………

**Questions related to Instagram**		

7	Do you use Instagram?	Yes ☐ No ☐
8	Do you have a personal account on Instagram?	Yes ☐ No ☐
9	How long have you been using Instagram?	Less than a year ☐ 1 to 3 years ☐ 3 to 5 years ☐ More than 5 years ☐
10	Approximately how long do you use Instagram daily?	Less than 2 h ☐ Between 2–4 h ☐ More than 4 h ☐ I don’t know ☐
11	Do you think the educational content available on Instagram are evidence based?	Yes ☐ No ☐ I don’t know ☐
12	If a professor suggests an Instagram account for educational purposes, will you follow it?	Yes ☐ No ☐ I don’t know ☐
13	What is your most important reason for using Instagram? (choose one option only)	Communicating with friends and acquaintances ☐ Sharing educational sources ☐ Learning educational contents ☐ Exchanging opinions with other experts ☐ For entertainment ☐ Job hunting ☐ Advertising ☐ Daily news ☐
14	What are your other reasons for using Instagram? (please number the desired items in order of usage)	Communicating with friends and acquaintances ☐ Sharing educational sources ☐ Learning educational contents ☐ Exchanging opinions with other experts ☐ For entertainment ☐ Job hunting ☐ Advertising ☐ Daily news ☐
15	Mostly from which part of Instagram do you get your educational content?	Posts containing only images ☐ Posts containing videos ☐ Story☐ IGTV ☐ Reels ☐ Hashtags ☐ I use them equally ☐
16	If captions under an educational post on Instagram were long, would you read it?	Yes, if it’s under 4 sentences ☐ Yes, if it’s over 4 sentences ☐ I don’t know ☐
17	If you saw uncredited scientific content on Instagram, do you search for its official source?	Yes ☐ No ☐
18	If you come across new scientific content (that you’re unfamiliar with) on Instagram, do you seek reputable sources to complete your information?	Yes ☐ No ☐
19	How much is the influence of the educational content on Instagram on your learning? (low influence: 1, high influence: 5)	1 ☐ 2 ☐ 3 ☐ 4 ☐ 5 ☐
20	What’s the most important disadvantage of using Instagram for educational purposes? (choose one option only)	The possibility of lack of focus and addiction to Instagram ☐ Difficulty in direct communication with information publishers and Q&A ☐ Time consuming to find credible sources ☐ Incompleteness of learning materials ☐
21	What are other important disadvantages of using Instagram for educational purposes? (please number the desired items in order)	The possibility of lack of focus and addiction to Instagram ☐ Difficulty in direct communication with information publishers and Q&A ☐ Time consuming to find credible sources ☐ Incompleteness of learning materials ☐

**Questions related to WhatsApp and Telegram**		

22	Do you use WhatsApp and telegram?	Yes, both ☐ Only WhatsApp ☐ Only Telegram ☐ No ☐
23	How long have you been using WhatsApp and Telegram?	Less than a year ☐ 1 to 3 years ☐ 3 to 5 years ☐ More than 5 years ☐
24	Approximately how much time do you spend on WhatsApp and Telegram daily?	Less than 2 h ☐ Between 2 and 4 h ☐ More than 4 h ☐ I don’t know ☐
25	Approximately how much time do you use WhatsApp and Telegram for educational purposes daily?	Less than 2 h ☐ Between 2 and 4 h ☐ More than 4 h ☐ I don’t know ☐
26	What is the main reason for your use of WhatsApp and Telegram? (choose one option only)	Communication with friends and acquaintances ☐ Sharing educational resources ☐ Learning educational content ☐ Exchanging ideas with other experts ☐ For entertainment ☐ Job hunting ☐ Advertising ☐ Daily news ☐
27	Other reasons for your use of WhatsApp and Telegram include which of the following? (number all applicable items in order)	Communication with friends and acquaintances ☐ Sharing educational resources ☐ Learning educational content ☐ Exchanging ideas with other experts ☐ For entertainment ☐ Job hunting ☐ Advertising ☐ Daily news ☐
28	From which section of Telegram do you receive most of your educational content?	Educational groups ☐ Educational channels ☐ I use both equally ☐
29	To what extent do the resources available on WhatsApp and Telegram influence your learning in a subject? (low influence: 1, High influence: 5)	1 ☐ 2 ☐ 3 ☐ 4 ☐ 5 ☐
30	The educational resources you use on WhatsApp and Telegram mostly consist of which type? Rate all options from 1 (most used) to 5 (least used).	Educational videos ☐ Lecture notes ☐ Source books ☐ Educational images ☐ Lecturer audios ☐
31	If you encounter scientific content without a source on WhatsApp and Telegram, do you search for its official source?	Yes ☐ No ☐
32	Do you think the educational resources provided on WhatsApp and Telegram are evidence based?	Yes ☐ No ☐ I don’t know ☐

**Questions related to YouTube**		

33	Do you use YouTube?	Yes ☐ No ☐
34	How long have you been using YouTube?	Less than a year ☐ 1 to 3 years ☐ 3 to 5 years ☐ More than 5 years ☐
35	How often do you use YouTube?	Daily ☐ Weekly ☐ Monthly ☐ yearly ☐
36	Approximately how much time do you spend on YouTube daily? (Only If you selected “Daily” in the previous question, answer this one.)	Less than 2 h ☐ Between 2 and 4 h ☐ More than 4 h ☐ I don’t know ☐
37	To what extent do the resources available on YouTube influence your learning in a subject? (low influence: 1, High influence: 5)	1 ☐ 2 ☐ 3 ☐ 4 ☐ 5 ☐
38	What are the reasons for your use of YouTube? (Number all items in order of priority.)	For entertainment ☐ Watching nonacademic instructional videos ☐ Watching academic instructional videos ☐
39	In your opinion, the videos available on YouTube are useful for learning practical courses in dentistry.	Strongly agree ☐ Agree ☐ Neutral ☐ Disagree ☐ Strongly disagree ☐
40	How likely are you to watch YouTube videos to prepare for practical work in dentistry that you haven’t done before?	I will definitely do this ☐ I am likely to do this ☐ I am unlikely to do this ☐ I will not do this at all ☐
41	Which of the following statements best describes your use of YouTube for learning practical courses in dentistry?	YouTube is my primary source of learning ☐ I use YouTube alongside university course materials to learn different techniques ☐ I use YouTube when I haven’t attended the desired class. ☐
42	Do you recommend using YouTube for learning practical courses in dentistry to your classmates?	Yes ☐ No ☐ Neutral ☐
43	Do you agree that your dental school should post instructional videos for learning practical courses in dentistry on YouTube?	Yes ☐ No ☐ Neutral ☐
44	For which practical courses in dentistry do you watch YouTube videos to gain the necessary readiness? (Check all that apply)	Complete denture ☐ Fixed prosthodontics ☐ Restorative dentistry ☐ Periodontology ☐ Endodontics ☐ Surgery: Tooth extraction ☐ Surgery: Suturing ☐ Orthodontics ☐ Pediatric dentistry ☐ Implantology ☐ Radiology ☐
45	In your opinion, are the instructional videos for practical courses in dentistry available on YouTube evidence based?	Yes ☐ No ☐ Neutral ☐
46	If you see scientific content without a source on YouTube, do you search for its official source?	Yes ☐ No ☐

**Final questions**		

47	Generally, which category of instructors do you think uses social media for education more?	Female instructors ☐ Male instructors ☐ No difference ☐
48	Generally, what age group of instructors use social media for education?	Mostly under 40 years old ☐ Mostly over 40 years old ☐ No difference ☐
49	Which social media do you prefer for learning academic contents?	Instagram ☐ WhatsApp and Telegram ☐ YouTube ☐
50	When using social media to aid in learning academic content, what priority do you give to each source? Rate from 1 (highest priority) to 6 (lowest priority).	Shorter study time or file viewing ☐ Simplicity of presented content ☐ Ability to use the source on a mobile device ☐ Up‐to‐date contents ☐ Credibility of information publisher ☐ Proper information classification ☐
51	In your opinion, what are the advantages of social media in learning? Prioritize from 1 to 3.	More diverse resources ☐ More engaging learning environment ☐ Increased creativity in teaching ☐
52	What influence have social media had on your learning during your studies? Please share your thoughts. (Optional question)	…………………………………………….. ……………………………………………… ………………………………………………
53	Do you use these social medias? (Select all that apply)	LinkedIn ☐ Twitter ☐ Facebook ☐ TikTok ☐
54	Which method of education do you prefer?	Only online classes ☐ Online classes alongside in‐person classes ☐ Only in‐person classes ☐ No preference ☐
55	Have you ever used these educational platforms? (Select all that apply)	Zoom ☐ Skype ☐ Skyroom ☐ Big Blue Button (3B) ☐
